# Use of an AI Scribe and Electronic Health Record Efficiency

**DOI:** 10.1001/jamanetworkopen.2025.37000

**Published:** 2025-10-10

**Authors:** Kevin Pearlman, Wen Wan, Sachin Shah, Neda Laiteerapong

**Affiliations:** 1 Department of Medicine, University of Chicago, Chicago, Illinois

## Abstract

**Importance:**

Time spent interacting with electronic health records (EHRs) is strongly associated with clinician burnout. Artificial intelligence (AI) scribes may offer a promising solution to EHR-related burnout. However, previous studies on their effectiveness are limited by selection bias.

**Objective:**

To evaluate the association of an AI scribe with EHR efficiency using a pre-post analysis among AI scribe users and a comparison of AI scribe users with a covariate-balanced control group of nonusers.

**Design, Setting, and Participants:**

This retrospective cohort study included ambulatory clinicians at an academic health system during a 3-month pilot period (July 1 to September 30, 2024).

**Exposure:**

Use of an AI scribe.

**Main Outcomes and Measures:**

Primary outcomes were time spent in the EHR, time spent in notes, and after-hours time spent documenting (“pajama time”) (all per appointment). Secondary outcomes were time to close encounters, appointment length, and monthly appointment volume. Two analyses were conducted: a within-individual pre-post comparison of AI scribe users (n = 125) and nonusers (n = 478), and a between-group comparison of AI scribe users and nonusers using propensity score overlap weighting to balance covariates.

**Results:**

A total of 125 AI scribe users (83 women [66.4%]; 69 [55.2%] with >10 years in practice; 46 [36.8%] in a medical subspecialty, 45 [36.0%] in surgery, and 34 [27.2%] in primary care) and 478 covariate-balanced AI scribe nonusers (267 women [55.9%]; 248 [51.9%] with >10 years in practice; 233 [48.7%] in a medical subspecialty, 155 [32.4%] in surgery, and 90 [18.8%] in primary care) were included. In the pre-post analysis, AI scribe users experienced significant reductions in median time in the EHR per appointment (baseline: median, 22.2 minutes [IQR, 12.1-37.0 minutes]; intervention period: median, 20.2 minutes [IQR, 11.5-31.4 minutes]; difference, −2.0 minutes; *P* < .001), time in notes per appointment (baseline: median, 7.5 minutes [IQR, 4.3-13.4 minutes]; intervention period: median, 7.0 minutes [IQR, 3.6-10.8 minutes]; difference, −0.5 minutes; *P* < .001), and time to close encounters (baseline: median, 24.4 hours [IQR, 7.7-94.0 hours]; intervention period: median, 17.3 hours [IQR, 5.4-57.0 hours]; difference, −7.1 hours; *P* < .001), with no significant differences in after-hours time spent documenting, appointment length, or appointment volume. In the weighted generalized linear regression, AI scribe use was associated with an 8.5% (95% CI, −12.8% to −3.9%; *P* < .001) lower mean EHR time (ie, 2.4 minutes) and a 15.9% (95% CI −21.2% to −10.4%; *P* < .001) lower mean time in notes (ie, 1.8 minutes) with no significant differences in other outcomes.

**Conclusions and Relevance:**

In this retrospective cohort study, clinicians using an AI scribe spent significantly less time in the EHR and in notes in both pre-post and propensity score analyses. These findings suggest that AI scribes may improve documentation efficiency and reduce clinician workload.

## Key Points

**Question:**

Is an ambulatory clinician’s use of an artificial intelligence (AI) scribe associated with improved efficiency in the electronic health record (EHR) when compared with covariate-balanced controls?

**Findings:**

In this cohort study including 125 AI scribe users and 478 covariate-balanced nonusers, clinicians who used an AI scribe had reductions in time spent in the EHR system and time in notes (per appointment) compared with the control group. No changes were identified in after-hours time spent documenting per appointment, mean time to close encounter, mean appointment length, or monthly number of completed office visits.

**Meaning:**

These findings suggest that AI scribes are associated with reductions in the amount of time clinicians spend documenting and writing notes in the EHR.

## Teaser

This cohort study assesses whether an ambulatory clinician’s use of an artificial intelligence (AI) scribe was associated with improved efficiency in the electronic health record when compared with covariate-balanced controls.

## Introduction

Electronic health record (EHR) documentation burden is a major source of clinician burnout in the US.^1,2^ Clinician burnout has been associated with higher turnover and lower quality of care.^3^ To alleviate EHR-related documentation burden, many health systems have historically turned to human scribes. Existing studies have shown that human scribes can positively affect efficiency metrics, including after-hours documentation (“pajama time”) and time spent on notes.^4,5,6,7,8,9^ However, the scalability of scribes is limited by training and personnel costs.^10^

Artificial intelligence (AI)–enabled scribes may offer a more scalable approach to alleviating clinical documentation burden. AI scribes use natural language processing to convert spoken interactions during clinical encounters into structured Subjective, Objective, Assessment, and Plan (SOAP) notes. Some observational studies suggest that AI scribes may reduce documentation time, while others have yielded more neutral results.^11,12,13,14,15,16^

A key limitation to most of these studies is their reliance on pre-post designs that lack control groups, which introduces potential selection bias. Clinicians who decide to use AI scribes may differ fundamentally from those who do not, as early adopters of new innovations tend to exhibit distinct characteristics.^17^ The few AI scribe studies that did include control groups did not completely address selection bias and had mixed results.^11,12^

To address this gap in the AI scribe literature, we conducted a retrospective cohort study using 2 complementary approaches: a pre-post analysis of both AI scribe users and nonusers to enable comparison with prior studies and a propensity score analysis comparing users and nonusers of the AI scribe to account for potential selection bias.

## Methods

### Setting

This study was conducted in the ambulatory setting at the University of Chicago Medical Center from July 1 to September 30, 2024. The University of Chicago Medical Center is an academic health system with roughly 1.3 million outpatient encounters annually. The study was determined to be quality improvement research by the University of Chicago Medical Center and was exempt from the need for institutional review board–mandated consent. Reporting adhered to the Strengthening the Reporting of Observational Studies in Epidemiology (STROBE) reporting guideline for cohort studies.

### AI Scribe Implementation

In July 2024, the University of Chicago deployed an AI scribe developed by Abridge AI Inc. The tool was offered to clinicians as a 3-month pilot. After obtaining patient informed consent, clinicians used their personal devices to audio record appointments with an Abridge AI–developed mobile application. After the visit, clinicians could review the AI-generated note for editing and transfer into the EHR. All University of Chicago ambulatory attending physicians and advanced practice professionals (APPs) were eligible to participate in the AI scribe pilot. The program was advertised via email and at department meetings. Participation was voluntary. Volunteers were required to complete a live 1-hour virtual training session led by Abridge AI technical support.

### Participants

For the analysis, clinicians were required to have complete baseline data for the variables used in the propensity score. Most missing data were associated with the absence of metrics for after-hours time spent documenting, which are not calculated for clinicians averaging 5 or fewer appointments per week. In addition, telehealth visits could not be used to calculate appointment length because of variable online check-in times; this limitation excluded a small number of clinicians. Finally, clinicians who enrolled in the AI scribe pilot but never used the software were excluded (see eFigure 1 in Supplement 1 for a flow diagram detailing clinician selection).

### Variables and Data Sources

The main outcomes were mean time in the EHR per appointment, mean time in notes per appointment, and mean after-hours time spent documenting per appointment (ie, time in EHR from 5:30 pm to 7 am on weekdays, weekends, and holidays). Other outcomes included mean time to close encounter, mean appointment length, and mean monthly appointment volume. Electronic health record use data were extracted from Epic’s Signal database for the 3-month pre-pilot baseline period (April 1 to June 30, 2024) and the 3-month pilot period (July 1 to September 30, 2024). Clinician location (hospital based vs offsite), type (physician vs APP), and specialty category (primary care, medical subspecialty, and surgical or procedural) were also extracted from Signal. Signal was also used to query each clinician’s Epic proficiency score, a proxy of EHR efficiency that evaluates the clinician’s use of certain functionalities (eg, EHR search, preference list use). Clinician sex and number of years since completing training were provided by the Medical Staff Office. Data regarding appointment length, time to close encounter, and appointment volume were provided by our institution’s Center for Research Informatics.

### Study Design

This study used 2 complementary designs. First, a pre-post study compared the outcomes for clinicians who did use the AI scribe with the outcomes for clinicians who did not use the AI scribe. Second, a propensity score–weighted analysis compared AI scribe users with nonusers during the pilot period.

### Statistical Analysis

For the pre-post analysis, we used Wilcoxon signed rank tests to assess changes for both pilot and control clinicians. We then evaluated primary outcomes across quartiles of AI scribe use.

For the propensity score analysis, we anticipated baseline imbalances between the pilot and control groups. Given the AI tool’s availability to all clinicians, we estimated the mean treatment effect among the entire population. To minimize the degree of model dependence in the statistical estimation of causal effects, we applied the overlap weighting method to balance baseline characteristics.^18,19^ The overlap weighting method mimics a randomized study by assigning weights to generate a target population that overlaps between groups. To assign these weights, propensity scores were estimated using logistic regression, representing the estimated probability of being an AI scribe user based on all given covariates. Each participant in the intervention group was assigned a weight equal to the probability of not receiving the treatment (ie, 1 − propensity score), while each participant in the control group received a weight equal to the probability of receiving the treatment (propensity score). As a result, individuals with overlapping propensity scores between the groups received a higher weight, while those in the nonoverlapping tails of the propensity score distribution received lower weights.

Before conducting a logistic regression for propensity score estimation, we checked for multicollinearity between baseline characteristics using Spearman correlation coefficients and variance inflation factors. For characteristics with high correlation (>0.9) and variance inflation factor (>5), only 1 variable was included. As such, time in the EHR per appointment was excluded due to its high correlation and variance inflation factor with time in notes per appointment.

Covariates used for overlap weighting included clinician sex, practice location, clinician type (physician vs APP), specialty, years in practice, and several preintervention workflow metrics: Epic proficiency score, time in notes per appointment, after-hours time spent documenting per appointment, mean time to close encounter, mean appointment length, and mean monthly completed appointments. Covariate balance was assessed using standardized mean differences.

After assigning weights to each participant, we used a weighted generalized linear model with a gamma distribution and log-link function to evaluate the intervention effect while adjusting for baseline characteristics and potential confounders. The gamma distribution was used because of the right-skewed distribution of the outcome variables. Given known disparities in EHR burden by sex and specialty, we also performed exploratory subgroup analyses stratified by sex, years in practice, specialty, clinician type (physician vs APP), and practice location (hospital based vs offsite).^20,21,22^

RStudio, version 2024.12.0 + 467 (R Project for Statistical Computing), was used for all statistical analysis. This research used ChatGPT 4.o (OpenAI) for assistance with coding, visual optimization of graphs, and refinement of manuscript text. All *P* values were from 2-sided tests and results were deemed statistically significant at *P* < .05.

## Results

A total of 603 clinicians were included in the final study cohort, comprising 125 AI scribe pilot participants (83 women [66.4%] and 42 men [33.6%]; 69 [55.2%] with >10 years in practice; 46 [36.8%] in a medical subspecialty, 45 [36.0%] in surgery, and 34 [27.2%] in primary care) and 478 control clinicians (267 women [55.9%] and 211 men [44.1%]; 248 [51.9%] with >10 years in practice; 233 [48.7%] in a medical subspecialty, 155 [32.4%] in surgery, and 90 [18.8%] in primary care) (Table 1). Pilot clinicians were predominantly physicians (117 of 125 [93.6%]) and worked largely in hospital-based clinics (81 of 125 [64.8%]). Preweighting differences between the pilot and control groups were largest for clinician sex, clinician type, Epic proficiency score, time to close encounters, and monthly appointment volume. After balancing, all standardized mean differences were less than 0.1, indicating an adequate balance (Table 1). Propensity score distributions before and after weighting are shown in eFigure 2 in Supplement 1.

**Table 1.** Baseline Characteristics of Pilot and Control Groups (April-June 2024)^a^

Characteristic	Unweighted			Weighted		
	Pilot group (n = 125)	Control group (n = 478)	Difference	Pilot group (n = 125)	Control group (n = 478)	Difference
Sex, No. (%)						
Female	83 (66.4)	267 (55.9)	0.11	75.2 (62.8)	210.1 (62.8)	0.0
Male	42 (33.6)	211 (44.1)	−0.11	44.6 (37.2)	124.5 (37.2)	0.0
Years since completing training, No. (%)						
≤10	56 (44.8)	230 (48.1)	−0.03	55.4 (46.2)	154.7 (46.2)	0.0
11-20	40 (32.0)	115 (24.1)	0.08	34.6 (28.9)	96.7 (28.9)	0.0
>20	29 (23.2)	133 (27.8)	−0.05	29.8 (24.9)	83.1 (24.9)	0.0
Hospital-based clinic, No. (%)						
No	44 (35.2)	162 (33.9)	0.01	42.4 (35.4)	118.3 (35.4)	0.0
Yes	81 (64.8)	316 (66.1)	−0.01	77.4 (64.6)	216.3 (64.6)	0.0
Clinician type, No. (%)						
Advanced practice professional	8 (6.4)	108 (22.6)	−0.16	9.9 (8.3)	27.7 (8.3)	0.0
Physician	117 (93.6)	37 (77.4)	0.16	109.8 (91.7)	306.9 (91.7)	0.0
Specialty category, No. (%)						
Primary care	34 (27.2)	90 (18.8)	0.08	30.1 (25.1)	84.1 (25.1)	0.0
Medical subspecialty	46 (36.8)	233 (48.7)	−0.12	48.1 (40.2)	134.5 (40.2)	0.0
Surgical or procedural	45 (36.0)	155 (32.4)	0.04	41.6 (34.7)	116.1 (34.7)	0.0
EPIC Proficiency score, mean (SD)	4.8 (2.2)	3.7 (2.1)	0.50	4.5 (2.1)	4.5 (2.2)	0.0
Time in notes per appointment, mean (SD), min	11.2 (12.9)	12.2 (11.7)	−0.09	11.0 (10.5)	11.0 (11.7)	0.0
After-hours time spent documenting per appointment, mean (SD), min	5.3 (9.7)	4.7 (6.0)	0.07	4.9 (5.9)	4.9 (8.4)	0.0
Time to close encounter, mean (SD), h	85.9 (191.2)	61.6 (147.8)	0.13	77.4 (193.9)	77.4 (168.0)	0.0
Appointment length, mean (SD), h	1.5 (0.7)	1.5 (0.9)	0.03	1.5 (0.8)	1.5 (0.7)	0.0
No. of monthly completed appointments, mean (SD)	105.3 (86.3)	96.4 (78.5)	0.11	105.1 (83.2)	105.1 (84.9)	0.0

^a^ Unweighted and weighted comparisons are shown for sex, training, specialty category, user type, and key outcome variables. Differences represent standardized mean differences for categorical and continuous variables. After weighting, all standardized mean differences were less than 0.01.

During the pilot period, the AI scribe was used in 48.3% of total encounters (19 486 of 40 350) by the 125 participating clinicians. Use of the AI scribe varied substantially across individuals, with clinicians using the scribe in a median of 41.6% (IQR, 18.2%-80.3%) of their encounters.

### Pre-Post Analysis

Compared with the 3-month preperiod, both control and pilot clinicians had a lower median time in EHR per appointment (control baseline: median, 24.2 minutes [IQR, 14.6-37.4 minutes]; intervention period: median, 23.8 minutes [IQR, 13.7-36.0 minutes]; difference, –0.4 minutes; *P* = .001; pilot baseline: median, 22.2 minutes [IQR, 12.1-37.0 minutes]; intervention period: median, 20.2 minutes [IQR, 11.5-31.4 minutes]; difference, −2.0 minutes; *P* < .001). Compared with the 3-month preperiod, both control and pilot clinicians had a lower median time in notes per appointment (control baseline: median, 9.2 minutes [IQR, 4.8-16.3 minutes]; intervention period: median, 9.0 minutes [IQR, 4.5-14.7 minutes]; difference, –0.2 minutes; *P* < .001; pilot baseline: median, 7.5 minutes [IQR, 4.3-13.4 minutes]; intervention period: median, 7.0 minutes [IQR, 3.6-10.8 minutes]; difference, −0.5 minutes; *P* < .001) (Table 2). Control clinicians had a small increase in time to close encounters (baseline: median, 16.9 hours [IQR, 4.9-58.3 hours]; intervention period: median, 17.7 hours [IQR, 4.7-51.0 hours]; difference, 0.8 hours; *P* < .001), and pilot clinicians had a large decrease in time to close encounter (baseline: median, 24.4 hours [IQR, 7.7-94.0 hours]; intervention period: median, 17.3 hours [IQR, 5.4-57.0 hours]; difference, −7.1 hours; *P* < .001). No differences were observed in after-hours time spent documenting per appointment, appointment length, or monthly appointment volume.

**Table 2.** EHR Outcomes Before and After Artificial Intelligence Scribe Use^a^

Outcome	Pilot group (n = 125)					Control group (n = 478)				
	Clinicians with data, No. (%)	Median (IQR)		Change in median	*P* value	Clinicians with data, No. (%)	Median (IQR)		Change in median	*P* value
		Baseline	Intervention period				Baseline	Intervention period		
Time in EHR per appointment, min	122 (97.6)	22.2 (12.1 to 37.0)	20.2 (11.5 to 31.4)	−2.0	<.001	459 (96.0)	24.2 (14.6 to 37.4)	23.8 (13.7 to 36.0)	−0.4	.001
Time in notes per appointment, min	122 (97.6)	7.5 (4.3 to 13.4)	7.0 (3.6 to 10.8)	−0.5	<.001	459 (96.0)	9.2 (4.8 to 16.3)	9.0 (4.5 to 14.7)	−0.2	<.001
After-hours time spent documenting per appointment, min	113 (90.4)	3.2 (1.0 to 5.8)	2.7 (1.0 to 5.8)	−0.5	.95	431 (90.1)	2.6 (0.8 to 6.2)	2.5 (0.8 to 6.2)	−0.1	.47
Mean time to close encounter, h	123 (98.4)	24.4 (7.7 to 94.0)	17.3 (5.4 to 57.0)	−7.1	<.001	463 (96.9)	16.9 (4.9 to 58.3)	17.7 (4.7 to 51.0)	0.8	<.001
Mean appointment length, h	123 (98.4)	1.3 (1.1 to 1.6)	1.3 (1.0 to 1.5)	0	.64	457 (95.6)	1.2 (1.0 to 1.5)	1.2 (1.0 to 1.5)	0.0	.14
Monthly no. of completed appointments	123 (98.4)	73.3 (43.3 to 137.0)	76.7 (43.3 to 148.0)	3.3	.19	459 (96.0)	68.0 (43.4 to 126.0)	68.7 (42.3 to 123.0)	0.7	.08

Abbreviation: EHR, electronic health record.

^a^ *P* values were calculated using the Wilcoxon signed rank test for paired data. Outcome data were incomplete for some clinicians, primarily due to missing data on after-hours time spent documenting. This missingness largely reflected clinicians with low clinical volume during 1 or more pilot months (eg, ≤5 appointments per week), for whom our institution’s EHR vendor does not report after-hours time spent documenting.

**Figure 1.**
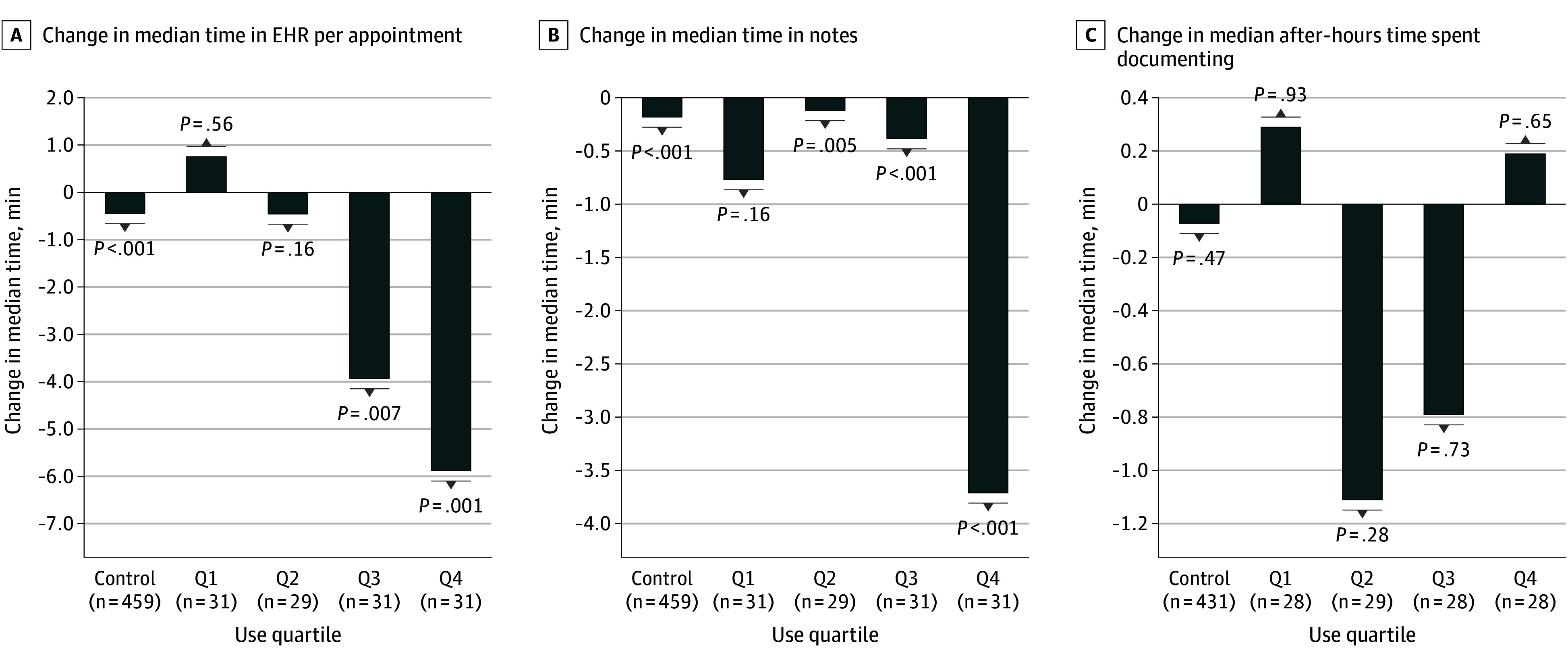
Pre–Artificial Intelligence (AI) vs Post-AI Scribe Primary Outcomes for Pilot Clinicians Stratified by Use Quartile (Q) vs Control Group Median change in total electronic health record (EHR) time, time in notes, and after-hours time spent documenting per appointment stratified by AI scribe use quartile. Each bar represents the difference between preimplementation and postimplementation median values. Quartiles reflect increasing scribe use (Q1 = lowest, Q4 = highest). The control group is included for reference. Comparisons were performed using the Wilcoxon signed rank test. Numbers of clinicians vary slightly for each outcome, as outcome data were incomplete for some clinicians, primarily due to missing data on after-hours time spent documenting. This missingness largely reflected clinicians with low clinical volume during 1 or more pilot months (eg, <5 appointments/week), for whom our institution’s EHR vendor does not report after-hours time spent documenting.

In the quartile analysis of the frequency of AI scribe use, more frequent use was associated with greater reductions in total EHR time and time in notes per appointment (Figure 1). Among clinicians in the top quartile of AI scribe use (Q4: >80% appointments using the AI scribe), there were significant within-group reductions in both time in EHR per appointment (baseline: median, 29.0 minutes [IQR, 15.0-38.8 minutes]; intervention period: median, 23.1 minutes [IQR, 16.8-40.8 minutes]; difference, −5.9 minutes; *P* < .001) and time in notes per appointment (baseline: median, 10.5 minutes [IQR, 5.8-14.8 minutes]; intervention period: median, 6.8 minutes [IQR, 4.5-10.9 minutes]; difference, −3.7 minutes; *P* < .001). The next highest quartile (Q3: 42%-80% of appointments using the AI scribe) demonstrated statistically significant reductions of lower magnitude for time in EHR per appointment (baseline: median, 24.7 minutes [IQR, 17.3-29.9 minutes]; intervention period: median, 20.8 minutes [IQR, 14.2-31.4 minutes]; difference, −3.9 minutes; *P* = .007) and time in notes per appointment (baseline: median, 7.8 minutes [IQR, 6.2-15.3 minutes]; intervention period: median, 7.4 minutes [IQR, 5.3-10.3 minutes]; difference, −0.5 minutes; *P* < .001, respectively). Quartile 2 (18%-42% of appointments using the AI scribe) demonstrated a small reduction in time in notes per appointment only (baseline: median, 7.8 minutes [IQR, 3.7-14.4 minutes]; intervention period: median, 7.7 minutes [IQR, 2.9-10.9 minutes]; difference, −0.1 minutes; *P* = .005). Quartile 1 (<18% of appointments using the AI scribe) did not have changes in these outcomes.

### EHR Efficiency Outcomes for Pilot vs Control Group

**Figure 2.**
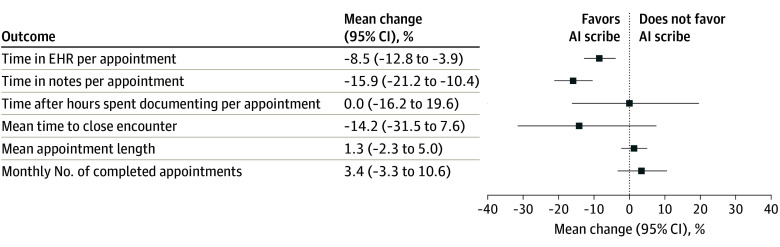
Artificial Intelligence (AI) Scribe Users vs Control Group by Outcome, July-September 2024 Percentage change in mean outcomes is based on exponentiated coefficients from generalized linear models using a log link and gamma distribution. All models were adjusted for sex, years since completing training, specialty category, clinician type, practicing at a hospital-based clinic vs offsite, Epic proficiency score, and the preperiod (baseline) value of each outcome. EHR indicates electronic health record.

In the weighted generalized linear regression model, AI scribe use was associated with an 8.5% (95% CI, –12.8% to –3.9%; *P* < .001) reduction in time in EHR (ie, 2.4 minutes) and a 15.9% (95% CI, –21.2% to –10.4%; *P* < .001) reduction in time in notes per appointment (ie, 1.8 minutes) for pilot clinicians compared with controls (Figure 2). After-hours time spent documenting per appointment, mean time to close encounter, mean appointment length, and monthly appointment volume did not differ between the pilot and control groups.

### EHR Efficiency Outcomes Stratified by Clinician Sex, Years in Practice, Specialty Category, Location, and Clinician Type

Subgroup analyses revealed differential outcomes for AI scribe use across certain clinician characteristics. Female clinicians experienced a 9.8% reduction in in EHR time per appointment (95% CI, –15.3% to –3.9%; *P* = .002) and an 18.4% reduction in time in notes per appointment (95% CI, –24.6% to –11.6%; *P* < .001), whereas male clinicians did not (Table 3). Clinicians with 11 to 20 years in practice had reductions in both EHR time (−15.0%; 95% CI, –23.4% to –5.7%; *P* = .003) and note time (−23.4%; 95% CI, –33.2% to –12.2%; *P* < .001), while clinicians in practice for greater than 20 years experienced an improvement only in EHR time (−7.3%; 95% CI, −13.4% to −0.7%; *P* = .03). Clinicians with 10 or fewer years in practice demonstrated no changes. Hospital-based clinicians had significant reductions in EHR time (–11.7%; 95% CI, –16.9% to –6.1%; *P* < .001) and time in notes (–21.7%; 95% CI, –27.8% to –15.1%; *P* < .001), while offsite clinicians did not demonstrate any changes. Primary care clinicians had a 9.7% reduction in EHR time (95% CI, −16.2% to −2.6%; *P* = .009) and a 15.8% reduction in note time (95% CI, −22.9% to −8.0%; *P* < .001). Medical subspecialists showed similar reductions: a 12.5% reduction in EHR time (95% CI, −18.5% to −6.0%; *P* < .001) and 18.4% less note time (95% CI, −25.2% to −11.0%; *P* < .001). Surgical or procedural clinicians had no significant changes. Electronic health record time improvements were present for both APPs (–14.1%; 95% CI, –21.6% to –5.9%; *P* = .002) and physicians (–7.2%; 95% CI, –11.9% to –2.3%; *P* = .004). For after-hours time spent documenting, only APPs (–24.7%; 95% CI, –42.9% to –0.7%; *P* = .047) and clinicians practicing at a hospital-based clinic (–15.9%; 95% CI, –28.4% to –1.1%; *P* = .04) had significant reductions.

**Table 3.** Stratified Associations of Artificial Intelligence Scribe Use With EHR Time Outcomes^a^

Subgroup	Time per appointment					
	In EHR		In notes		After-hours time spent documenting	
	Change in mean % (95% CI)	*P* value	Change in mean % (95% CI)	*P* value	Change in mean % (95% CI)	*P* value
Sex						
Female	−9.8 (−15.3 to −3.9)	.002	−18.4 (−24.6 to −11.6)	<.001	−10.3 (−25.4 to 7.9)	.25
Male	−5.1 (−11.2 to 1.4)	.12	−8.2 (−16.3 to 0.8)	.07	5.7 (−11.7 to 26.6)	.54
Years since completing training						
≤10	−3.1 (−8.9 to 3.1)	.31	−11.1 (−17.9 to −3.8)	<.001	−5.2 (−19.9 to 12.2)	.54
11 to 20	−15.0 (−23.4 to −5.7)	.003	−23.4 (−33.2 to −12.2)	<.001	−4.1 (−22.5 to 18.7)	.70
>20	−7.3 (−13.4 to −0.7)	.03	−9.1 (−18.2 to 1.1)	.08	8.4 (−13.6 to 36.0)	.47
Hospital-based clinic						
No	−2.4 (−9.5 to 5.4)	.54	−5.8 (−14.8 to 4.1)	.24	17.5 (−1.7 to 40.5)	.08
Yes	−11.7 (−16.9 to −6.1)	<.001	−21.7 (−27.8 to −15.1)	<.001	−15.9 (−28.4 to −1.1)	.04
Clinician type						
Advanced practice professional	−14.1 (−21.6 to −5.9)	.002	−16.9 (−25.0 to −7.8)	<.001	−24.7 (−42.9 to −0.7)	.05
Physician	−7.2 (−11.9 to −2.3)	.004	−15.5 (−21.1 to −9.5)	<.001	−4.7 (−17.7 to 10.5)	.53
Specialty category						
Primary care	−9.7 (−16.2 to −2.6)	.009	−15.8 (−22.9 to −8.0)	<.001	−12.4 (−29.4 to 8.6)	.23
Medical subspecialty	−12.5 (−18.5 to −6.0)	<.001	−18.4 (−25.2 to −11.0)	<.001	−4.9 (−21.1 to 14.6)	.60
Surgical or procedural	−2.5 (−10.5 to 6.3)	.57	−10.0 (−20.1 to 1.4)	.08	16.2 (−3.3 to 39.7)	.11

Abbreviation: EHR, electronic health record.

^a^ Each subgroup analysis compares pilot clinicians with control clinicians with the same characteristic (eg, male pilot clinicians vs male controls) using overlap weighting. Percentage change in mean values and 95% CIs were derived from generalized linear models with a log link and gamma distribution. A separate model was fit for each subgroup. All models were adjusted for baseline (preperiod) values of the outcome and the other covariates used in the weighting model.

## Discussion

In this cohort study, we compared clinicians who participated in a 3-month AI scribe pilot with a control group of nonparticipating clinicians using both pre-post and propensity score analyses. In the propensity score analysis, we identified reductions in total EHR time and time in notes per appointment for the pilot group compared with controls. These findings were generally consistent with the pre-post analysis, which varied based on the frequency of AI scribe use.

Similar to findings regarding human scribes, our study found that the use of an AI scribe was associated with improvements in EHR efficiency.^4,5,6,7,8,9,23,24,25^ Unlike human scribes, however, AI scribes require no training and are not subject to turnover, both of which can carry financial and operational implications.^10^ As a more scalable alternative, AI scribes may help address longstanding EHR-associated burnout.^14,15,16^

Although our study did not demonstrate a reduction in after-hours EHR use or the time to close clinical encounters in the propensity score analysis, the observed decreases in total EHR time and time spent in notes suggest that clinicians were able to reallocate time during clinic hours to nondocumentation-related activities. Although a median reduction of 2.4 minutes in EHR time per appointment may appear modest in isolation, its clinical implications are meaningful when scaled across a typical clinic schedule. If a clinician sees 20 patients over the course of the day, the potential time savings amounts to 48 minutes—close to an hour that could be redirected toward direct patient care, engagement with clinical decision support tools, trainee education, or administrative tasks. This potential benefit may even exceed EHR time savings recently reported in a Stanford study, which demonstrated a 20-minute reduction in daily EHR time for clinicians using an AI scribe.^15^ Such efficiency gains may ultimately alleviate cognitive burden, as suggested in recent qualitative studies.^13,26^

Although encounter closure time decreased significantly in the pre-post analysis among AI scribe users (median reduction of 7.1 hours), this outcome was not statistically significant in the propensity score analysis. However, the propensity score analysis still favored the AI scribe group, suggesting a potential benefit that did not reach statistical significance due to limited power and high variability.

Our results suggest that female clinicians were more likely than their male counterparts to experience EHR efficiency gains with an AI scribe. This difference may be partially explained by female clinicians spending more time than male clinicians documenting at baseline.^21,22^ Furthermore, medical subspecialists and primary care clinicians experienced EHR efficiency gains while surgical specialists did not, which aligns with prior research on human scribes.^7^ Overall, the lack of improvement in certain subgroups may reflect a floor effect, whereby AI scribes offer limited benefit to clinicians who already spend minimal time documenting.

### Limitations

Our study has several limitations. Although we used propensity score overlap weighting to mitigate selection bias, some residual bias may remain. For example, participants may exhibit an “early adopter” phenotype, making them more inclined to embrace new technologies—a characteristic that may not have been captured by the available covariates. Another limitation is the imprecise measurement of EHR use. These measures do not differentiate between active computer engagement and periods when the EHR is open but not directly engaged with, such as during face-to-face patient encounters. They also cannot distinguish between ambulatory and nonambulatory EHR use (eg, inpatient consultation time). These other activities may lead to an underestimation of the EHR time reductions for outpatient ambulatory encounters. Our study also lacked patient-level data such as medical complexity and social determinants of health, which could affect documentation burden; thus, residual confounding from unmeasured patient differences remains a limitation. In addition, clinicians at our institution have long had access to a speech-to-text dictation tool (Dragon), and use of this technology was neither measured nor restricted during the study. Although we cannot rule out the use of speech-to-text tools as a potential confounder, they are unlikely to explain the observed reductions in EHR time—this technology has been available for years, and no changes in its promotion occurred during the study period. In addition, generalizability is limited to English-spoken encounters, as the AI scribe did not support non-English encounters. Furthermore, while AI scribes show promise in improving documentation efficiency, several studies have highlighted risks such as decreased note accuracy and increased note length.^13,26,27^ Measures to identify and correct these shortcomings can and should be pursued by vendors, clinicians, and health systems alike. Finally, AI’s application in health care is still in its early stages. As this technology continues to evolve, it will likely acquire additional functionalities that will affect clinician efficiency such as EHR summarization, order entry, visit diagnosis assignment, and clinical decision support.

## Conclusions

In this cohort study of clinicians using an AI scribe, significant reductions in total EHR time and time in notes were evident in both pre-post and propensity score analyses. Encounter closure time significantly improved in pre-post analyses among AI scribe users but did not differ significantly when compared with controls in the propensity score analysis. The greatest efficiency gains were observed among female clinicians, primary care clinicians, and medical subspecialists. No changes were identified in after-hours time spent documenting, appointment length, or appointment volume. These findings suggest that AI scribes may improve documentation efficiency and reduce clinician workload.

## Notes

Supplement 1. **eFigure 1.** Clinician Selection for AI Scribe Pilot Analysis Flow Diagram **eFigure 2.** Propensity Score Distributions of Pilot and Control Groups Before and After Covariate Balancing

Supplement 2.
Data Sharing Statement

## References

[1] Kroth PJ, Morioka-Douglas N, Veres S, . Association of electronic health record design and use factors with clinician stress and burnout. JAMA Netw Open. 2019;2(8):e199609. doi:10.1001/jamanetworkopen.2019.9609 31418810 PMC6704736

[2] Li C, Parpia C, Sriharan A, Keefe DT. Electronic medical record–related burnout in healthcare providers: a scoping review of outcomes and interventions. BMJ Open. 2022;12(8):e060865. doi:10.1136/bmjopen-2022-060865 35985785 PMC9396159

[3] Hodkinson A, Zhou A, Johnson J, . Associations of physician burnout with career engagement and quality of patient care: systematic review and meta-analysis. BMJ. 2022;378:e070442. doi:10.1136/bmj-2022-070442 36104064 PMC9472104

[4] Gidwani R, Nguyen C, Kofoed A, . Impact of scribes on physician satisfaction, patient satisfaction, and charting efficiency: a randomized controlled trial. Ann Fam Med. 2017;15(5):427-433. doi:10.1370/afm.2122 28893812 PMC5593725

[5] Micek MA, Arndt B, Baltus JJ, . The effect of remote scribes on primary care physicians’ wellness, EHR satisfaction, and EHR use. Healthc (Amst). 2022;10(4):100663. doi:10.1016/j.hjdsi.2022.100663 36375356

[6] Piersa AP, Laiteerapong N, Ham SA, . Impact of a medical scribe on clinical efficiency and quality in an academic general internal medicine practice. BMC Health Serv Res. 2021;21(1):686. doi:10.1186/s12913-021-06710-y 34247600 PMC8272908

[7] Rotenstein L, Melnick ER, Iannaccone C, . Virtual scribes and physician time spent on electronic health records. JAMA Netw Open. 2024;7(5):e2413140. doi:10.1001/jamanetworkopen.2024.13140 38787556 PMC11127114

[8] Lowry C, Orr K, Embry B, . Primary care scribes: writing a new story for safety net clinics. BMJ Open Qual. 2017;6(2):e000124. doi:10.1136/bmjoq-2017-000124 29435506 PMC5699154

[9] Elton AC, Schutte D, Ondrey G, Ondrey FG. Medical scribes improve documentation consistency and efficiency in an otolaryngology clinic. Am J Otolaryngol. 2022;43(4):103510. doi:10.1016/j.amjoto.2022.103510 35636088

[10] Miksanek TJ, Skandari MR, Ham SA, . The productivity requirements of implementing a medical scribe program. Ann Intern Med. 2021;174(1):1-7. doi:10.7326/M20-0428 33017564

[11] Haberle T, Cleveland C, Snow GL, . The impact of nuance DAX ambient listening AI documentation: a cohort study. J Am Med Inform Assoc. 2024;31(4):975-979. doi:10.1093/jamia/ocae022 38345343 PMC10990544

[12] Liu TL, Hetherington TC, Dharod A, . Does AI-powered clinical documentation enhance clinician efficiency? a longitudinal study. NEJM AI. Published online November 22, 2024. doi:10.1056/AIoa2400659

[13] Duggan MJ, Gervase J, Schoenbaum A, . Clinician experiences with ambient scribe technology to assist with documentation burden and efficiency. JAMA Netw Open. 2025;8(2):e2460637. doi:10.1001/jamanetworkopen.2024.60637 39969880 PMC11840636

[14] Cao DY, Silkey JR, Decker MC, Wanat KA. Artificial intelligence–driven digital scribes in clinical documentation: pilot study assessing the impact on dermatologist workflow and patient encounters. JAAD Int. 2024;15:149-151. doi:10.1016/j.jdin.2024.02.009 38571698 PMC10988030

[15] Ma SP, Liang AS, Shah SJ, . Ambient artificial intelligence scribes: utilization and impact on documentation time. J Am Med Inform Assoc. 2025;32(2):381-385. doi:10.1093/jamia/ocae304 39688515 PMC11756633

[16] Tierney AA, Gayre G, Hoberman B, . Ambient artificial intelligence scribes to alleviate the burden of clinical documentation. NEJM Catalyst. 2024;5(3):CAT.23.0404. doi:10.1056/CAT.23.0404

[17] Rogers EM. *Diffusion of Innovations*. 3rd ed. Macmillan Publishing Co Inc; 1983:453.

[18] Li F, Morgan K, Zaslavsky A. Balancing covariates via propensity score weighting. J Am Stat Assoc. 2018;113(521):390-400. doi:10.1080/01621459.2016.1260466

[19] Li F, Thomas LE, Li F. Addressing extreme propensity scores via the overlap weights. Am J Epidemiol. 2019;188(1):250-257. 30189042 10.1093/aje/kwy201

[20] Holmgren AJ, Sinsky CA, Rotenstein L, Apathy NC. National comparison of ambulatory physician electronic health record use across specialties. J Gen Intern Med. 2024;39(14):2868-2870. doi:10.1007/s11606-024-08930-4 38980460 PMC11534958

[21] Rotenstein LS, Fong AS, Jeffery MM, . Gender differences in time spent on documentation and the electronic health record in a large ambulatory network. JAMA Netw Open. 2022;5(3):e223935. doi:10.1001/jamanetworkopen.2022.3935 35323954 PMC8948526

[22] Malacon K, Touponse G, Yoseph E, . Gender differences in electronic health record usage among surgeons. JAMA Netw Open. 2024;7(7):e2421717. doi:10.1001/jamanetworkopen.2024.21717 39042410 PMC11267410

[23] Heckman J, Mukamal KJ, Christensen A, Reynolds EE. Medical scribes, provider and patient experience, and patient throughput: a trial in an academic general internal medicine practice. J Gen Intern Med. 2020;35(3):770-774. doi:10.1007/s11606-019-05352-5 31808131 PMC7080913

[24] Pozdnyakova A, Laiteerapong N, Volerman A, . Impact of medical scribes on physician and patient satisfaction in primary care. J Gen Intern Med. 2018;33(7):1109-1115. doi:10.1007/s11606-018-4434-6 29700790 PMC6025675

[25] Walker K, Ben-Meir M, Dunlop W, . Impact of scribes on emergency medicine doctors’ productivity and patient throughput: multicentre randomised trial. BMJ. 2019;364:l121. doi:10.1136/bmj.l121 30700408 PMC6353062

[26] Shah SJ, Crowell T, Jeong Y, . Physician perspectives on ambient AI scribes. JAMA Netw Open. 2025;8(3):e251904. doi:10.1001/jamanetworkopen.2025.1904 40126477 PMC11933996

[27] Biro J, Handley JL, Cobb NK, . Accuracy and safety of AI-enabled scribe technology: instrument validation study. J Med Internet Res. 2025;27:e64993. doi:10.2196/64993 39869899 PMC11811668

