# Supplementary material for: Use of an AI Scribe and Electronic Health Record Efficiency
Source: JAMA Netw Open. 2025 Oct 10;8(10):e2537000. doi: 10.1001/jamanetworkopen.2025.37000 (PMC12514625; doi:10.1001/jamanetworkopen.2025.37000)
Supplement: Supplement 1. — eFigure 1. Clinician Selection for AI Scribe Pilot Analysis Flow Diagram eFigure 2. Propensity Score Distributions of Pilot and Control Groups Before and After Covariate Balancing [file jamanetwopen-e2537000-s001.pdf]

Supplementary Online Content

Pearlman K, Wan W, Shah S, Laiteerapong N. Use of an AI scribe and electronic health record efficiency. *JAMA Netw Open.* 2025;8(10):e2537000.
doi:10.1001/jamanetworkopen.2025.37000

eFigure 1. Clinician Selection for AI Scribe Pilot Analysis Flow Diagram

eFigure 2. Propensity Score Distributions of Pilot and Control Groups Before and After Covariate Balancing

This supplementary material has been provided by the authors to give readers additional information about their work.

© 2025 Pearlman K et al. *JAMA Network Open.*

eFigure 1. Clinician Selection for AI Scribe Pilot Analysis Flow Diagram

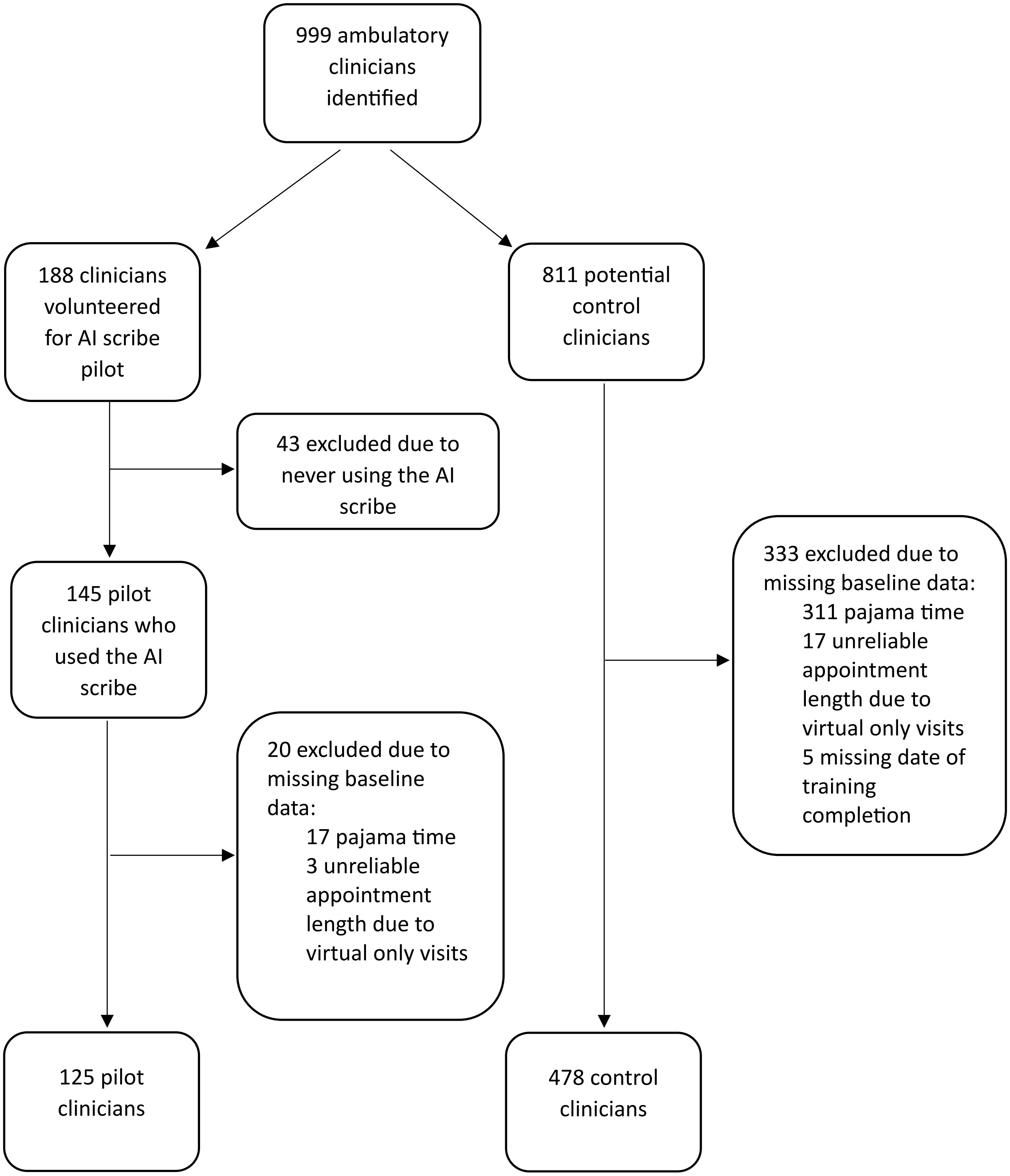

© 2025 Pearlman K et al. *JAMA Network Open.*

eFigure 2. Propensity Score Distributions of Pilot and Control Groups Before and After Covariate Balancing

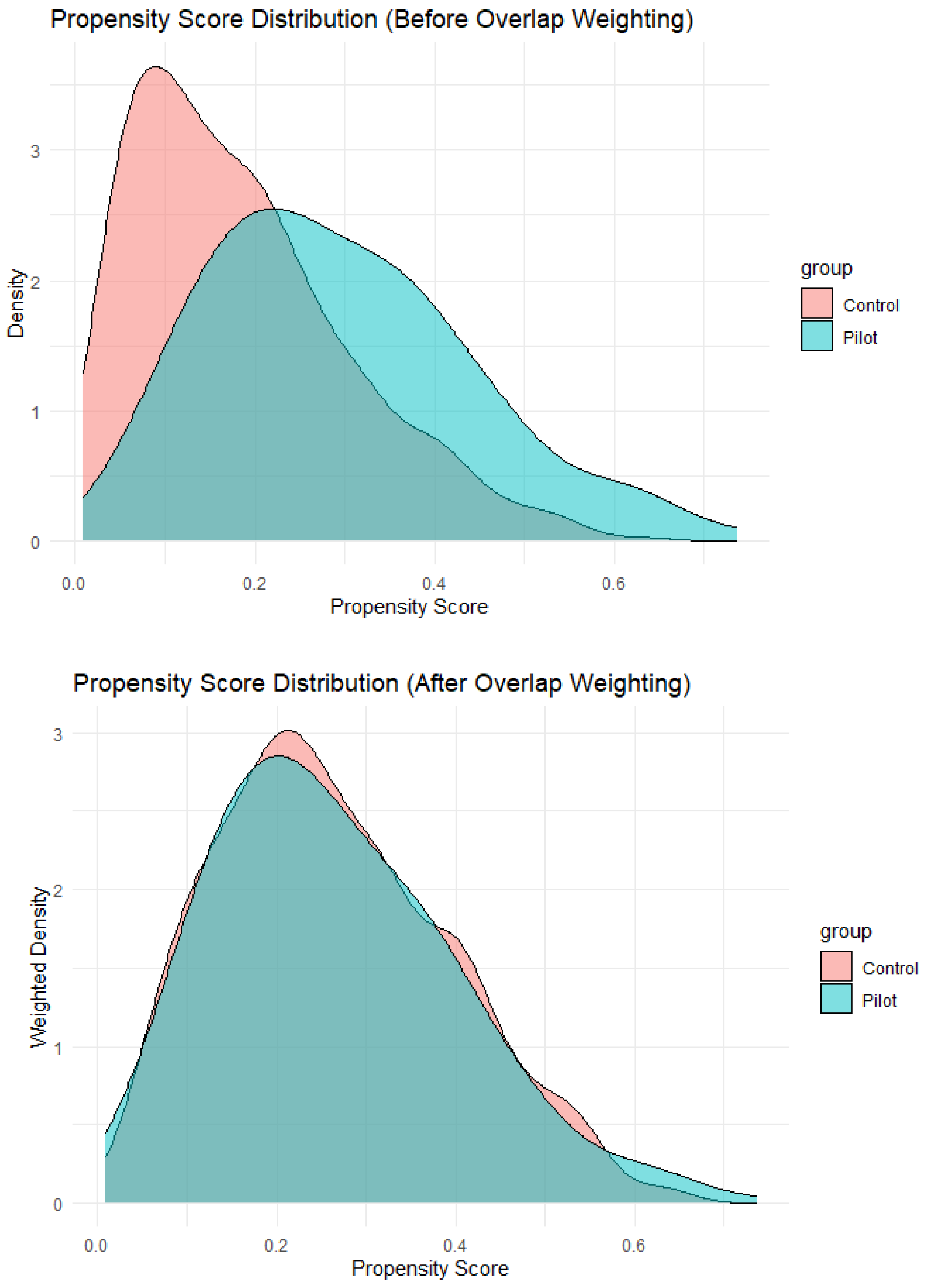

© 2025 Pearlman K et al. *JAMA Network Open.*
