# Supplementary material for: Use of an AI Scribe and Electronic Health Record Efficiency
Source: JAMA Netw Open. 2025 Oct 10;8(10):e2537000. doi: 10.1001/jamanetworkopen.2025.37000 (PMC12514625; doi:10.1001/jamanetworkopen.2025.37000)
Supplement: Supplement 2. — Data Sharing Statement [file jamanetwopen-e2537000-s002.pdf]

Data Sharing Statement

Pearlman. Use of an AI Scribe and Electronic Health Record Efficiency. *JAMA Netw Open*. Published October 10, 2025. doi:10.1001/jamanetworkopen.2025.37000

Data
Data available: No

Additional Information
Explanation for why data not available: Data is proprietary
